# A Case of Cyanosis

**Published:** 1881-11

**Authors:** M. H. Sears

**Affiliations:** Leadville, Colorado


### ARTICLE V.

### A CASE OF "CYANOSIS." By M. H. SEARS, M.D., Leadville, Colorado.

R. B., æt. five and one half months, was the child of parents apparently healthy. The father, a man of about thirty-three years of age, is free from tubercular or syphilitic taint, and temperate. The mother also free from taint, and previous to the birth of this child primaparous, is twenty-five years of age.

She states that the child was born at term with the aid of forceps—applied in the superior strait—after a labor of thirty-six hours duration. The child was apparently well developed, weighing about eight pounds, and made efforts to nurse during the succeeding twenty-four hours. The mother suffered from depressed nipples, and had very little milk to offer the child, so that its endeavors proved partially futile—the bottle being resorted to, to supply the deficiency. The mother, however, persevered in her endeavors to nurse her child, but at the end of about ne month desisted, and resorted to the bottle entirely as a means of its subsistence. Upon this diet it seemed for a time to thrive, but finally each of the artificial "foods for infants" was tried, only to be succeeded by other diets, none of which were successful.

When the child was about two weeks old, a peculiar bluish hue was noticed over the entire integumentary surface, and particularly was it noticed when the child was excited, as in crying. Nothing was thought of this symptom for the time being, but as this "cyanotic hue" increased and became very noticeable over the scalp, it was the subject of remark.

With the appearance of the hue the digestive function partially failed, and a crop of furuncles, varying in size from one-eighth to one-half an inch in diameter made themselves manifest. These were lanced, and poured forth a quantity of semi-decomposed pus, often tinged with blood. The bowels moved from two to six times in the twenty-four hours, and presented upon examination quantities of mucus and some fecal matter,—nothing, however, of value for diagnostic use.

At the time these conditions were noted, the child was four months old, and at this time it came under my observation. I found it very much emaciated, weighing less than it did at its birth, and vomiting within a very short time after anything was introduced into the stomach.

Upon physical examination the lungs revealed the normal vesicular murmur, the præcordia normal in its boundaries, as nearly as I could judge, as the child was "pigeon breasted;" the heart-sounds normal, except the sound occurring at the pulmonic orifice; this was at times wholly suppressed. Care was taken not to confound the sound of the aortic with that of the pulmonary when the latter was present. The aortic, on the other hand, was always normal and never gave evidence of being compromised in auscultation. With these facts before me, combined with the external symptoms, I made the provisional diagnosis "cyanosis." I immediately took steps to see if the error in the nutrition could be corrected, and prescribed a watery extract of beef prepared in the following manner:

One pound of lean beef finely chopped; one quart of cold water.

The beef was macerated in the water for six hours, strained, and the remaining liquid concentrated to one pint by boiling.

Of this extract, a teaspoonful was given every half hour. In conjunction with this diet, a powder consisting of bis. subnit. grs. v; and hyd. chlo. mite gr. ss, was given morning and evening. Under this plan of treatment the weight of the patient increased, the nausea and vomiting partially stopped, the stools became normal in color and consistency, and I had great hopes that I had adopted a plan destined to at least nourish the patient. But in time this failed, and I had to order milk, which I took pains to see was obtained from one animal, young, new pasture fed, and well cared for.

To each quart of this milk, liq. calcis ʒ ij. was added and the child allowed to nurse it from the bottle,—the milk previous to being introduced into the bottle was warmed. Nourishing enemata were also given and retained; his diet was carried out with varying success during the continuance of the case. Acid. hydroch. was tried very successfully, as an anti-emetic, in place of the bismuth salt, but its depressing action on the heart did not lead to its general use in the treatment of the case, although it proved itself fully able to check the distressing nausea and vomiting.

With these measures, the patient continued to improve slowly, but an unlooked for attack of cholera infantum aggravated all of the previous symptoms; and notwithstanding appropriate treatment was pursued to check it, the patient died in an asthenic condition. At the *post mortem* the following were the essential conditions:

The heart normal in size, general appearance, and situation; aorta, vena innominata and pulmonaries normal; ductus arteriosus impervious. An examination of the interior of the heart revealed the foramen ovale unclosed, and the Eustachian valve about as it would be in fœtal life—the perviousness of the foramen being the essential pathological condition.

Another point noted was the condition of the walls of the ventricles. The wall of the right was so thin and fragile as to be torn very easily. This probably arose from partial non-use of the ventricle, on account of much of the blood passing through the foramen causing a partial atrophy. The walls of the left ventricle on the other hand were large, very thick—muscular hypertrophy from over-use; the valves normal apparently. At the time of death, child was six months old lacking five days.

---

THE *Atlanta Medical and Surgical Journal* will be issued in future as the "*Atlanta Medical Register.*"

H. H. DICKSON, Publisher.

